# Ultrasensitivity in Phosphorylation-Dephosphorylation Cycles with Little Substrate

**DOI:** 10.1371/journal.pcbi.1003175

**Published:** 2013-08-08

**Authors:** Bruno M. C. Martins, Peter S. Swain

**Affiliations:** SynthSys – Synthetic and Systems Biology, The University of Edinburgh, Edinburgh, United Kingdom; Johns Hopkins University, United States of America

## Abstract

Cellular decision-making is driven by dynamic behaviours, such as the preparations for sunrise enabled by circadian rhythms and the choice of cell fates enabled by positive feedback. Such behaviours are often built upon ultrasensitive responses where a linear change in input generates a sigmoidal change in output. Phosphorylation-dephosphorylation cycles are one means to generate ultrasensitivity. Using bioinformatics, we show that *in vivo* levels of kinases and phosphatases frequently exceed the levels of their corresponding substrates in budding yeast. This result is in contrast to the conditions often required by zero-order ultrasensitivity, perhaps the most well known means for how such cycles become ultrasensitive. We therefore introduce a mechanism to generate ultrasensitivity when numbers of enzymes are higher than numbers of substrates. Our model combines distributive and non-distributive actions of the enzymes with two-stage binding and concerted allosteric transitions of the substrate. We use analytical and numerical methods to calculate the Hill number of the response. For a substrate with 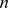 phosphosites, we find an upper bound of the Hill number of 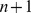, and so even systems with a single phosphosite can be ultrasensitive. Two-stage binding, where an enzyme must first bind to a binding site on the substrate before it can access the substrate's phosphosites, allows the enzymes to sequester the substrate. Such sequestration combined with competition for each phosphosite provides an intuitive explanation for the sigmoidal shifts in levels of phosphorylated substrate. Additionally, we find cases for which the response is not monotonic, but shows instead a peak at intermediate levels of input. Given its generality, we expect the mechanism described by our model to often underlay decision-making circuits in eukaryotic cells.

**Authors Summary**

Dose-response curves are said to be ultrasensitive when they are sigmoidal rather than hyperbolic and often underlay cellular decision-making circuits. Zero-order ultrasensitivity is a well-known mechanism to generate sigmoidal curves in phosphorylation cycles, but one of its assumptions often implies that the substrate is more abundant than the modifying enzymes. We show that this assumption is unlikely to always hold *in vivo*, and we present a general model that generates ultrasensitivity when the enzymes are in excess of their substrate. The model combines conformational allosteric transitions of the substrate with two-stage binding of the enzymes: the enzymes bind first to a docking site on the substrate and then to the substrate's phosphosites. Ultrasensitivity is generated because the kinase can bind to the fully phosphorylated form of the substrate (at its docking site) and sequester the substrate away from the phosphatase and, similarly, the phosphatase can bind to the fully dephosphorylated form of the substrate and sequester the substrate away from the kinase. The number of kinase-phosphatase competitions for the substrate determines the degree of ultrasensitivity. Finally, we show that this model can generate non-monotonic responses that peak at intermediate levels of input.

## Introduction

Covalent modifications, such as phosphorylations, play a pivotal role in regulating the activity of proteins and in the processing of extracellular signals in eukaryotic cells [1]. These modifications typically affect the tertiary structure [2] of the targeted proteins, thus changing their enzymatic activity or their binding affinities for other proteins. For phosphorylations, there are two types of modifying enzymes: kinases phosphorylate; phosphatases dephosphorylate. Cycles of phosphorylation-dephosphorylation are embedded in regulatory and signalling pathways and can regulate the flow of information inside the cell. Extracellular and intracellular cues are sensed and transduced to variations in the concentration of active enzymes. In the presence of a signal, an increase in activity of a kinase can, for example, activate a transcription factor and so turn on a programme of gene expression. The same programme of expression can be turned off by an increase in the activity of a phosphatase relative to the activity of the kinase. Phosphorylation cycles can therefore be treated as a mechanism to generate a dose-response curve or more generally an input-output relationship. The input is typically either the concentration of one of the enzymes (assuming the levels of the other enzyme are approximately constant on the time scale of the response) or the ratio of the concentration of one enzyme to the other. The output is a function of the phosphorylation state of the substrate.

Phosphorylation cycles have been predicted to exhibit ultrasensitivity, generating a response to a change in the stimulus that is more non-linear, or more sigmoidal, than a Michaelis-Menten-like response [3]. Ultrasensitivity, and the degree of non-linearity it implies, is important because it is a pre-requisite for biochemical networks to generate oscillations or bistable behaviour [4]. Sequential cascades of phosphorylation cycles, such as cascades of MAP kinase, can amplify signals and increase the degree of ultrasensitivity of the response with each step of the cascade [5]. Single cycles but with substrates with multiple phosphorylation sites can have steep response curves too, although the degree of ultrasensitivity can depend on the order in which the sites are modified and whether the modifying enzyme is processive or distributive [6], [7]. Product-inhibition of the kinase and phosphatase, in which their products remain bound to the enzymes [8], and other forms of sequestration [9], [10] also induce ultrasensitive behaviour.

Perhaps the most famous means, though, is zero-order ultrasensitivity [3], but this mechanism imposes constraints on the concentrations of enzymes and their substrates that do not always appear to hold *in vivo* [11]. Both enzymes must be saturated – the concentration of the substrate should far exceed the enzyme's Michaelis-Menten constant – so that the modifying reactions proceed at a rate that is independent of the concentration of the substrate. Such saturation often occurs when the concentration of the substrate is much greater than the concentration of the enzymes. Examples of zero-order ultrasensitivity are known *in vitro* [12], [13] and *in vivo* [14], [15], but other ultrasensitive systems have also been discovered with enzymes that are more abundant than the substrate [16].

Attempts at reconciling these opposing observations have attracted some modelling. Mechanisms to explain how an excess of enzymes over the substrate can still generate an ultrasensitive output have included product-inhibition [8] and local saturation [16], [17]. These models, however, require assumptions of their own or *ad hoc* constraints that affect their generality.

Here we estimate the distribution of enzymes-to-substrate ratios in the yeast *Saccharomyces cerevisiae* and so estimate how widespread are the conditions that invalidate zero-order ultrasensitivity. We then present a general, mechanistic model for robust ultrasensitivity in single and multi-site phosphorylation-dephosphorylation cycles in conditions where the enzymes are in excess relative to their substrate. We explore this novel mechanism, obtaining analytical calculations of the Hill number (a measure of ultrasensitivity), identify the origins of ultrasensitivity, and show that the mechanism can also exhibit non-monotonic responses.

## Results

### Distribution of saturation levels in phosphorylation reactions in budding yeast

The zero-order model of ultrasensitivity [3] hinges on the assumption that the enzymes are saturated and so enzymes to substrate ratios, 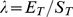, are often low, where 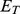 and 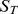 are the concentrations of enzyme and of substrate available in the system. Enzyme saturation can always occur when that ratio is sufficiently below one (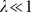).

Systematic measurements of Michaelis-Menten constants and the concentrations of enzymes and their substrates *in vivo* are still limited. Experimental evidence suggests that many enzymes operate away from saturation with ratios of substrate concentration to Michaelis-Menten constants ranging between 10^−2^ and 1 in physiological conditions [11]. We expanded on these reports and estimated the ratios of enzymes to substrate *in vivo* in the yeast *S. cerevisiae* by comparing information about phosphorylation reactions with global measurements of protein expression. We extracted from BioGRID [18], a curated database of protein-protein interactions, all interactions that are annotated as corresponding to either phosphorylations or dephosphorylations in budding yeast. We thus compiled a list of kinases and phosphatases and their respective substrates (which may themselves be kinases or phosphatases). We then compared these proteins with the protein expression data of Ghaemmaghami *et al.* [19], who constructed an extensive fusion library and used immunodetection to measure the absolute levels of protein abundance during log phase growth in rich media.

**Figure 1:**
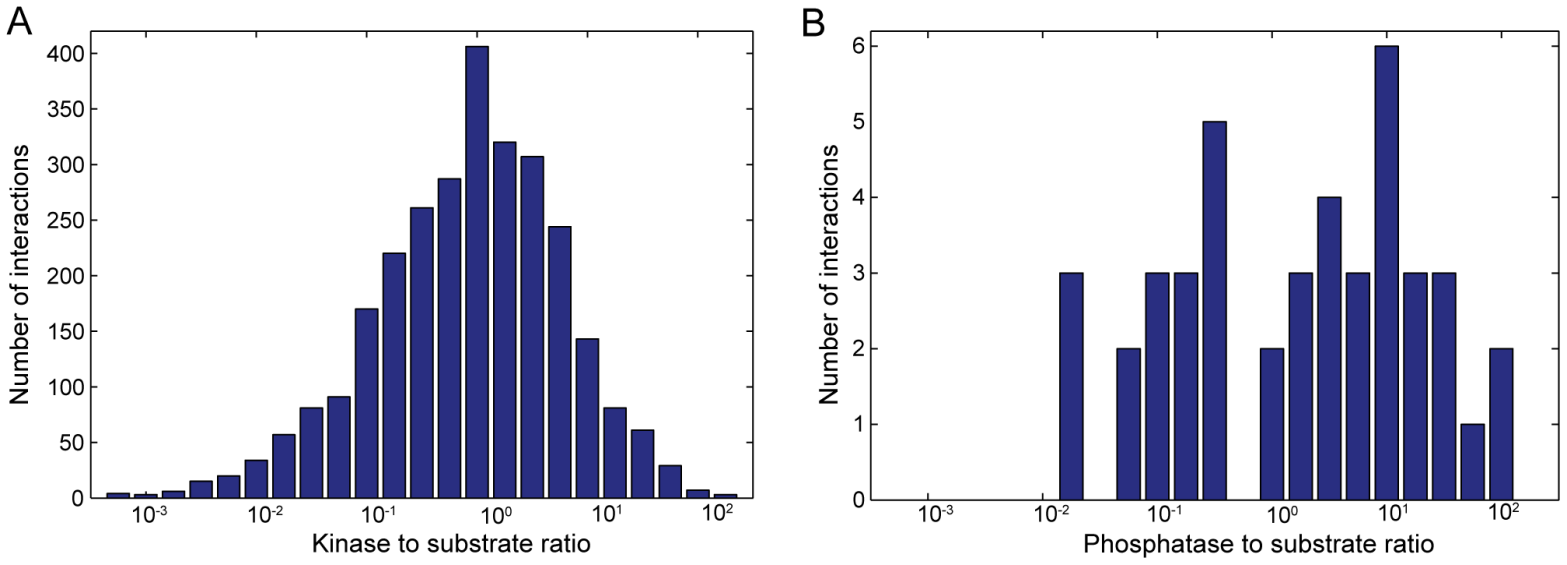
The ratio of the concentration of kinases to the concentration of their substrates does not appear biased in *S. cerevisiae*. (**A**) The distribution of kinase saturation levels, 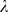 (the ratio of kinase concentration to substrate concentration), for the phosphorylation reactions in the BioGRID database [18] with levels of gene expression during log phase growth in rich media measured by Ghaemmaghami *et al.* [19]. The data contains 2850 phosphorylation reactions, comprising 98 unique kinases and 1136 unique substrate targets. (**B**) Equivalent distribution for phosphatases. The data contains 43 dephosphorylation reactions, comprising 16 unique phosphatases and 32 unique targets.

Taken together, these two sets of data allow us to show that the enzyme to substrate ratio, 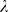, for a number of phosphorylation dependent systems in yeast does not appear to be biased (Fig. 1). We obtain 2850 phosphorylation reactions, comprising 98 unique kinases and 1136 unique substrates and 43 dephosphorylation reactions involving 16 unique phosphatases and 32 unique substrates. The data for phosphatases is scarce and thus not as informative, and so we focus on kinases. The distribution of ratios for kinases is unimodal, centred on a 1∶1 kinase to substrate ratio, with a minimum of 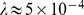 and a maximum of 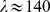 (Fig. 1). Half of all reactions (49%) operate under 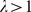 and so are likely not to satisfy the conditions for zero-order ultrasensitivity. It is worth noting that randomly sampling any two genes in the Ghaemmaghami *et al.* data set and calculating their ratio produces a distribution that is statistically different from Fig. 1A (Kolmogorov-Smirnov test), but is equally unimodal with a peak at approximately the same location. We therefore conclude that a distribution of 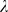 that is significantly different from a baseline distribution has not been selected, and so ultrasensitivity in phosphorylation cycles ought not to be a phenomenon that depends on extreme values of concentrations.

This observation prompted us to develop a model that shows ultrasensitivity in phosphorylation cycles when 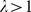.

### The model

Our model combines concerted allosteric transitions of the state of the substrate [20] with an implicit form of two-stage binding.

A covalent modification of a substrate molecule often throws a conformational switch that modifies the molecule's tertiary structure [2], [21]. Conformational changes are naturally described by allosteric transitions [20], [22]–[26]. We assume that the substrate alternates between active and inactive states through thermal fluctuations and may be biased towards one or another state by its level of phosphorylation. A related model with conformational changes controlled by phosphorylation reactions has been used previously to describe the nuclear translocation of a family of transcription factors of the immune system [27].

**Figure 2:**
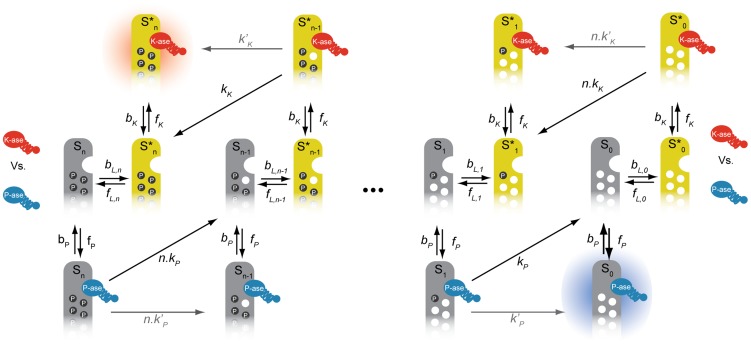
A model of a phosphorylation-dephosphorylation cycle for an allosteric substrate with multiple phosphorylation sites has two “sink” states and multiple competitions between the modifying enzymes for the substrate. For each level of phosphorylation, the free substrate switches conformations allosterically between 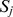 (inactive, in grey) and 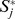 (active, in yellow). The rate of allosteric transitions from active states to inactive states is 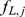 and the reverse rate of allosteric transitions from inactive states to active states is 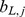. The kinase (in red) binds to the active forms of the substrate; the phosphatase (in blue) binds to the inactive forms. The two sinks 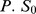 and 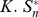, are shown with shadows. The rate of association of the kinase to the substrate is 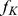; the rate of dissociation of the kinase-substrate complex is 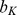. Phosphorylation of a phosphosite resulting in dissociation of the kinase from the substrate has rate 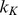 per phosphosite (distributive catalytic rate represented by the diagonal black arrows). Phosphorylation of a phosphosite whereby the enzyme remains bound to the substrate has a rate 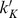 (non-distributive catalytic rate represented by the straight grey arrows). The rate of association of the phosphatase to the substrate is 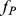 and its rate of dissociation is 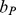. De-phosphorylation of a phosphosite resulting in dissociation of the phosphatase from the substrate has a distributive rate 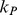 per phosphosite. Dephosphorylation of a phosphosite whereby the enzyme remains bound to the substrate has a non-distributive rate 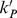.

In a two-stage binding mechanism, the enzymes – the kinase and the phosphatase – bind to their target in two independent but sequential steps: first, they bind to a docking site on the substrate molecule; second, and only subsequently, are the enzymes able to find, bind to and catalyze the modification of a phosphosite, perhaps through a rearrangement of the substrate's tertiary structure [28], [29]. We assume that the kinase can only bind to its docking site on the substrate when the substrate is in its active form and that the phosphatase can only bind to its docking site when the substrate is inactive. Both enzymes may either use the same docking site (or partly overlapping docking sites) or different docking sites, but in either case, the structural conformation of the active state is such that the phosphatase cannot bind and the structural conformation of the inactive state is such that the kinase cannot bind. Two-stage binding implies that the kinase can still bind a molecule of substrate when the substrate's phosphosites are all phosphorylated and, likewise, the phosphatase can still bind the substrate when the substrate's phosphosites are all unphosphorylated because each enzyme can always bind to their docking site (Fig. 2).

The output or response of a phosphorylation cycle is typically considered to be a function of the phosphorylation state of the substrate, but there is no consensus on what this function should be. With only a single phosphosite, the fraction of phosphorylated substrate, either free or in complex with one of the two enzymes, can be used. When there are multiple phosphosites and so multiple states of partial phosphorylation, there is no longer a unique choice of output. Some studies have used the fraction of the fully phosphorylated substrate [6], but it seems likely that substrates could have some activity when only partially phosphorylated. Others have taken an average of the phosphorylation states, weighting by the number of phosphorylated sites for each individual molecule [17], but proteins are perhaps unlikely to have such tightly regulated patterns of activity. The level of output could also depend on which sites are phosphorylated [30] or depend only on a few essential sites [31], but these choice require systems that are well characterized experimentally. Our allosteric mechanism avoids this dilemma: each molecule must be either active or inactive and the response function is the fraction of substrate that is active [27].

Our model therefore works as follows (Fig. 2): substrates with an arbitrary number of phosphosites, 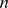, undergo conformational transitions between active forms, 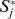, and inactive forms, 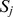, where 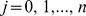 is the number of phosphorylated sites on each molecule. The enzyme-substrate complexes that form are 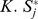 and 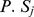, where 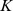 denotes the kinase and 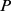 denotes the phosphatase. Only then can each enzyme modify the phosphorylation state of a single phosphosite. Both enzymes compete for the free form of the substrate at each stage of phosphorylation, which allosterically transitions between the two states 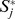 and 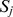. The competition for a particular molecule of substrate does not therefore occur at the same time, but still exists because of each molecule switching back and forth between the forms preferred by each enzyme (Fig. 2). Further, the enzyme-substrate complex may either dissociate following each modification – in which case the enzymatic reactions follow a distributive scheme [32] – or remain bound after each modification – in which case the catalytic reactions may follow a processive scheme [33]. By formulating a model where both distributive and processive schemes can coexist, we can analyse their contributions to the degree of ultrasensitivity in the system.

We note that it is not strictly necessary to assume, as we have here, that distributive phosphorylation and dephosphorylation reactions trigger the dissociation of the enzyme-substrate complex. It is mechanistically more likely that there is an intermediate complex, and it is from this complex that the enzymes dissociate to subsequently rebind and catalyse the modification of the remaining phosphosites distributively. This extra layer does not, however, introduce significant qualitative changes [16], thus in the interest of simplicity and mathematical tractability we consider only a single step for the interaction of enzymes with the modifiable phosphosites, the catalytic modification of the phosphosites and the dissociation of the enzyme-substrate complex in the distributive case (Fig. 2).

The model can be described by a system of ordinary differential equations, where the phosphatase binds the substrate at rate 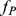, dissociates from the substrate at rate 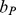, and catalyses the dephosphorylation of the substrate at a distributive rate 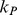 or at a non-distributive rate 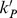. The rates 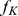, 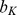, 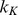 and 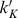 are defined similarly for the kinase. The dynamics of the enzyme-substrate complexes, remembering the phosphatase cannot bind the substrate in the active state and the phosphatase cannot bind the substrate in the inactive state, obey:
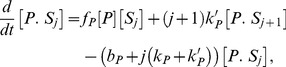
(1)

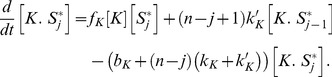
(2)The positive terms in Eqs. (1) and (2) describe the formation of enzyme-substrate complexes through binding of free enzymes to their docking sites in the substrate or through non-distributive enzymatic reactions. The negative terms describe the destruction of the enzyme-substrate complexes through unbinding of the enzymes from their docking sites in the substrate or through either distributive or non-distributive enzymatic reactions. We assume all phosphosites are identical and equally available to be bound and modified by the enzymes. Therefore the catalytic rates of dephosphorylation 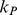 and 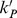 are multiplied by the number of phosphorylated phosphosites that are available to the phosphatase at each state. Likewise, the catalytic rates of phosphorylation 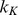 and 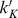 are multiplied by the number of unphosphorylated phosphosites that are available to the kinase at each state.

The dynamics of the active and inactive free forms of the substrate – where 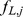 is the rate of the conformational transition of the substrate from active to inactive states, and can depend in principle on the number 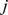 of phosphorylated phosphosites, and 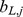 is the rate for transitions from inactive to active substrate states – is described by:
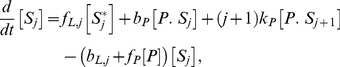
(3)

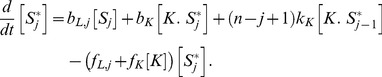
(4)The positive terms in Eqs. (3) and (4) describe the production of free forms of the substrate either through allosteric transitions from free forms of the opposite state, through dissociation of the enzyme-substrate complexes, or through distributive enzymatic reactions. The negative terms describe the destruction of free forms of the substrate either through allosteric transitions to free forms of the opposite state or through binding of free enzymes to their docking sites in the substrate.

Finally, the dynamics of the free forms of the enzymes obeys
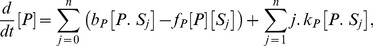
(5)

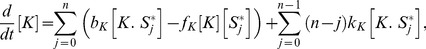
(6)where 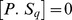 and 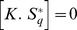 if 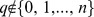. The positive terms in Eqs. (5) and (6) describe the release of enzymes from the substrate either through unbinding of the enzymes from their docking sites in the substrate or through distributive enzymatic reactions. The negative terms describe the binding of free enzymes to their docking sites in the substrate.

We normalise all concentrations by the total concentration of substrate, 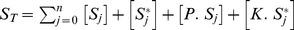, and impose that 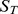 and the total amount of kinase and phosphatase, 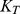 and 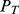, are conserved (enzymatic modifications occur faster than gene expression and protein degradation). In these units, 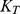 is therefore equal to 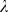. We solve Eqs. (1–4) at steady state to obtain:
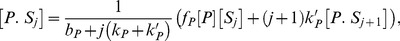
(7)

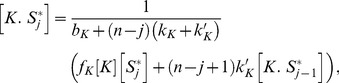
(8)

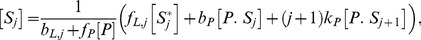
(9)

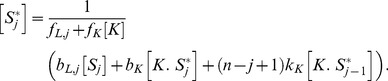
(10)The output of the system is the fraction of active substrate
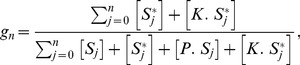
(11)and 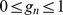. The input is the ratio of the concentrations of free enzymes [3]. We assume that the kinase is maintained at a constant concentration, but that phosphatase varies in concentration, either due to activation through signaling or to a change in cellular location. For example, the phosphatase Ptc1 is recruited to its substrate at the plasma membrane in response to increasing concentrations of input (extracellular pheromone) during mating in yeast [16]. Finally, and because we are interested in regimes where the concentration of the enzymes is several fold higher than that of the substrate, we can treat the concentration of free enzymes to be approximately the same as their total concentration (the right hand side of Eqs. (5) and (6) is approximately zero). We write the approximation as 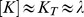, because all concentrations are normalised to the concentration of substrate in our model.

### Analytical calculation of the upper bound of the Hill number

The Hill number is a commonly used measure of ultrasensitivity and is defined as [34],
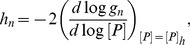
(12)where 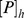 is the concentration of phosphatase that generates an activity that is halfway between the maximum and minimum activities (the IC50). The basal and minimal levels of activity are, respectively,
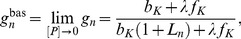
(13)

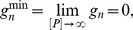
(14)where 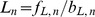 is the allosteric equilibrium constant, and 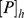 obeys 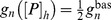.

In the symmetric case, we can analytically find the Hill number and can show that it has a maximum of 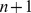. We simplify our calculations (without affecting the general conclusion) by letting both enzymes operating at identical rates, i.e., 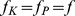, 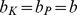, 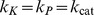, 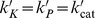.

We will start by exploring the scenario where all allosteric rates are identical, 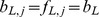, and therefore the allosteric equilibrium constant 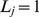. The modification of the state of the substrate by the enzymes does not then bias the equilibrium of the free forms of substrate towards a state favoured by one or the other enzyme (for the kinase, the active state; for the phosphatase, the inactive state). We obtain the general solution
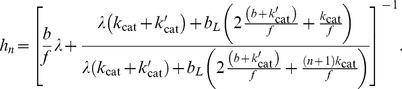
(15)If we assume that the allosteric reactions 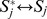 are faster than all other reactions (i.e., 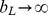), so that the allosteric transitions are close to equilibrium, then Eq. (15) becomes
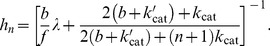
(16)
Eq. (16) has an ultrasensitive maximum of 
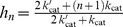
 (when 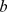 – the dissociation rate of the enzyme-substrate complexes – is much smaller than the other rates) and a subsensitive minimum of 0. Purely distributive schemes of phosphorylation-dephosphorylation (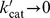) can therefore generate a maximum Hill number of 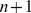.

In this regime of fast and non-biased allosteric kinetics, non-distributive schemes of phosphorylation-dephosphorylation (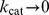) are not ultrasensitive as they can only attain a Hill number of at most 1 (Eqs. (15) and (16)). Non-distributive systems may, however, be ultrasensitive if one assumes the existence of product-inhibition, i.e., if the affinity of the kinase to its docking sites in the substrate is much enhanced when the substrate is phosphorylated (and likewise the affinity of the phosphatase is enhanced when the substrate is dephosphorylated) [8]. Here we assume interactions of the enzymes with the docking site are independent of the number and state of phosphosites.

For a system with 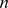 phosphosites, numerical simulations with 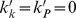 and randomly sampled (non-symmetric) parameters confirm that the Hill number is distributed between 0 and 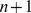 (Fig. S1). The general conditions necessary to generate ultrasensitivity when the allosteric transitions are unbiased by the state of the substrate (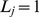) are therefore: distributive enzymatic reactions, fast allosteric kinetics and stability of the enzyme-substrate complex. If the enzyme-substrate complex is unstable (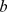 is the dominant rate), the system is subsensitive.

The maximum Hill number is given by the number of competitions between the kinase and the phosphatase for the substrate (Fig. 2). This result implies that even systems with substrates with a single phosphosite can generate ultrasensitive responses with Hill numbers of up to 2. For 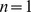, for example, the enzymes compete twice: for the substrate states 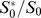 and 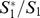.

### Ultrasensitivity is caused by sequestration of the substrate in the sink states 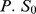 and 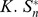


**Figure 3:**
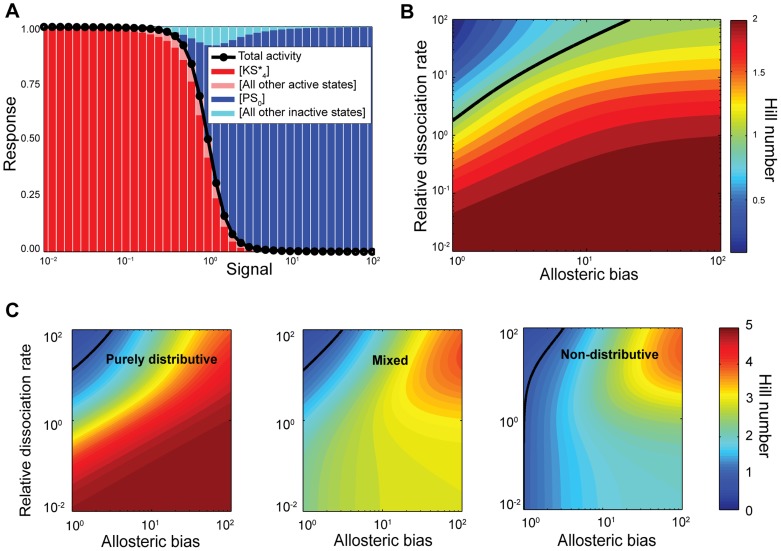
Ultrasensitivity occurs because of sequestration of the substrate into one of two sinks (either fully phosphorylated and bound to kinase or fully dephosphorylated and bound to phosphatase) and is enhanced by the existence of an allosteric bias. (**A**) Simulation of the response curve (in black) and the concentration of the states of the system (coloured bars) as a function of the signal, i.e., the ratio of phosphatase to kinase. Here we have 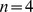, 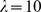, 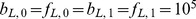 s^−1^, 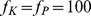 s^−1^ (in units of the inverse total concentration of the substrate), 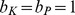 s^−1^, 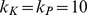 s^−1^, 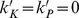. The Hill number is approximately 3.5. (**B**) Contour plot of the Hill number as a function of the allosteric bias and the relative dissociation rate obtained from Eq. (17). In this panel 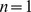, the allosteric bias is given by 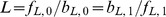 and the relative dissociation rate is 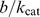 with 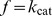 and 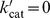. **(C) An allosteric bias allows non-distributive systems to become ultrasensitive.** Contour plots of the Hill number as a function of the allosteric bias and the relative dissociation rate, obtained from solving Eq. (12) for 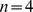. Left: purely distributive case (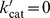); centre: coexistence of distributivity and non-distributivity (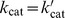); right: non-distributive case (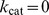). The relative dissociation rate is defined as in (B) and 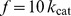, except for the right panel where the relative dissociation rate is defined as 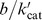 and 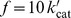. In (B) and (C), the solid black line marks the boundary between subsensitivity (above the line) and ultrasensitivity (below the line).

The ultrasensitive input-output curve is dominated by the concentrations of mainly two states. Fig. 3A depicts an example of one such ultrasensitive curve, with the concentration of some of the states at each level of input also shown. The response curve follows closely the variation in the concentration of the states 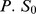 and 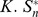. These states are the complexes formed by the binding of the phosphatase to the fully unphosphorylated substrate and by the binding of the kinase to the fully phosphorylated substrate. Both states exist because of two-stage binding and the binding of the enzyme to its docking site. Although each enzyme cannot modify the phosphorylation state of the substrate further, by remaining bound, the enzyme still sequesters a molecule of substrate from the competing enzyme.

Ultrasensitivity is therefore generated by sequestration, which is enabled by two-stage binding. When there is little phosphatase in the system (low signal in Fig. 3A), the kinase operates freely, phosphorylates the substrate almost completely and then remains bound to substrate in the state 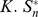 (Fig. 2). Most of the substrate will be sequestered by the kinase because the kinase is more abundant than the substrate (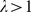), and, as determined above, a condition for ultrasensitivity is a small dissociation rate of the enzyme-substrate complex. As the phosphatase enters and accumulates in the system, it has little impact initially because most of the substrate is shielded from it and the amount of free kinase is still enough to win the competition for the little free substrate that is available. As more phosphatase is added, it eventually starts winning the competition for the substrate, and once the phosphatase can win one competition then it can win all 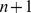, and so sequesters the substrate in its own sink, 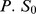 (Fig. 2). The concentration of 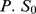 rises sharply as 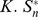 collapses (Fig. 3A). When the ratio of phosphatase to kinase is high (high input) the situation has been completely reversed and the substrate is almost completely sequestered by the phosphatase and beyond the reach of the kinase. The existence of the two sink states, 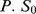 and 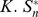, therefore makes ultrasensitivity possible in a phosphorylation cycle with 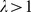. It is indeed a necessary condition that the enzymes are in excess relative to the substrate because only then they can sequester the substrate entirely.

### Modulation of the allosteric bias by the enzymes enhances ultrasensitivity

While we conclude from Eq. (16) that the enzymes need not modulate the allosteric conformational transitions of the substrate for the system to be ultrasensitive, the higher the allosteric bias induced by each enzyme in that enzyme's favour the more ultrasensitive the system becomes. If the free substrate is more biased towards the active form when it is more phosphorylated, then this bias favours binding of the kinase because the kinase binds only to active substrate. Similarly, if the free substrate is more biased towards the inactive form when it is less phosphorylated, then this bias favours binding of the phosphatase because the phosphatase binds only to inactive substrate. Such an induced bias could also be necessary if, for example, there is promiscuity and so crosstalk between different enzymes and substrates from various subsystems. Enzymes that modulate the allosteric bias of their substrate can force the substrate into conformational states that prevent other enzymes from physically binding. For 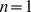, and assuming symmetry, we impose the bias by setting 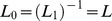, where 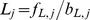 is the allosteric equilibrium constant. From Eq. (12) and, taking the limit of fast allosteric rates, we obtain
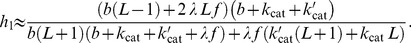
(17)
Eq. (17) has the same upper bound of 2, i.e., 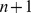, but shows enhanced ultrasensitivity, all other rates being equal (Fig. 3B).

The presence of an allosteric bias can generate ultrasensitivity even if one of the conditions we presented above for the non-biased case – the stability of enzyme-substrate complexes – does not hold. In such cases, while the sink state 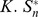 may not be effective in sequestering the substrate away from the phosphatase, the allosteric bias increases the probability of the fully phosphorylated substrate being in the active state, which the phosphatase cannot access, thus preventing linear changes in the overall activity in response to linear changes in the levels of the phosphatase (Fig. S3).

Additionally, allosteric biases can drive non-distributive systems, where 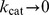, into ultrasensitivity. From Eq. (17), a moderate degree of ultrasensitivity is reached when the dissociation constant of the enzymes-substrate complex is low (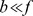) or when the rate of dissociation is much slower than the catalytic rate (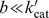) and the allosteric bias of the substrate is high (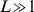). Under these conditions, the system becomes processive: the enzymes bind to their docking site and proceed to phosphorylate or dephosphorylate all phosphosites with few dissociation events. Eventual dissociation of the kinase is therefore most likely to occur only when the substrate is fully phosphorylated, and eventual dissociation of the phosphatase is most likely to occur only when the substrate is fully dephosphorylated. The Hill number can approach an upper limit of 2, which is independent of the number of phosphosites. As discussed before, two-stage binding means the enzymes can still bind to the docking site even if no phosphosites are available for modification; effectively, the enzymes therefore compete twice for the free forms of the substrate.

Higher Hill numbers occur when the non-distributive catalytic rate is much slower than both the binding rate of the enzymes to their docking site and the dissociation rate (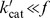 and 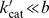) and the allosteric bias is high (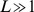). Under these conditions, the enzymes are more likely to unbind from their docking site and dissociate from the substrate following each enzymatic reaction than to modify the next phosphosite. Consequently, both enzymes may compete for the free forms of the substrate at each state of phosphorylation, and the upper bound of the Hill number is therefore 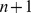. These conditions make the system quasi-distributive, but the degree of ultrasensitivity is substantially constrained when compared to an equivalent system that is purely distributive (Fig. 3C and Figs. S4–S6).

### Non-monotonic response curves

**Figure 4:**
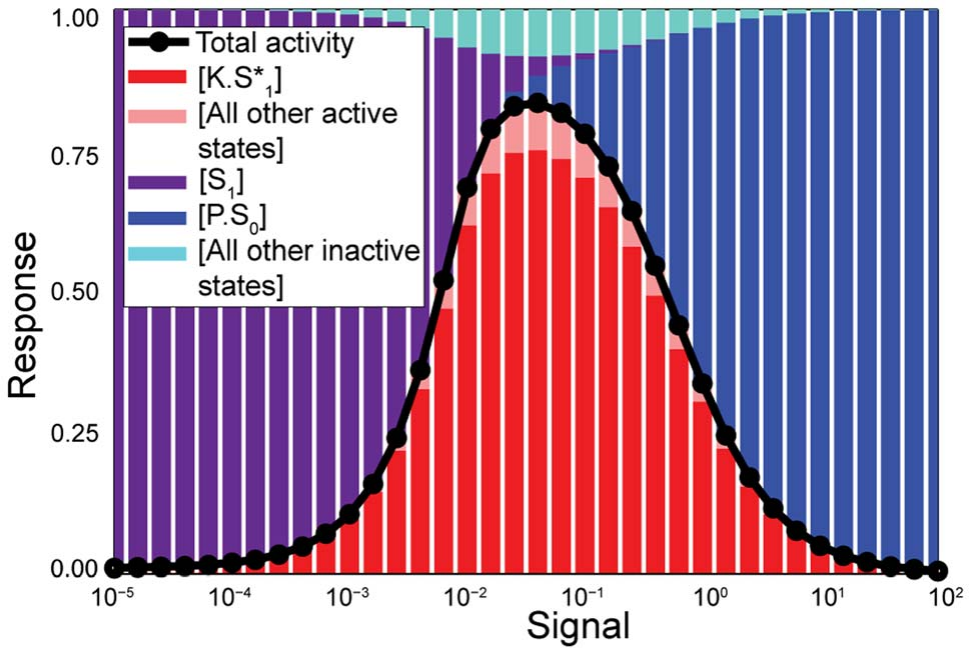
Phosphorylation-dephosphorylation cycles can generate non-monotonic input-output relationships. Here the allostery of the substrate inhibits further modifying reactions by disfavouring binding of the kinase when the substrate is phosphorylated and disfavouring binding of the phosphatase when the substrate is dephosphorylated. Simulation of the response curve (in black) and the concentration of the states of the system (coloured bars) as a function of the signal, i.e., the ratio of phosphatase to kinase, for a system where 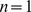, 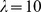, 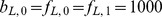 s^−1^, 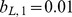 s^−1^, 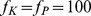 s^−1^ (in units of the inverse total concentration of the substrate), 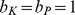 s^−1^, 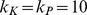 s^−1^, 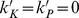.

When, perhaps counter-intuitively, the fully phosphorylated state induces a strong conformational bias towards the inactive state, and thus hinders the binding of kinase, the system can generate a non-monotonic response with a peak of activity at intermediate levels of input (Fig. 4). If only kinase is present in the system (low input), the activity is low because there is a strong bias towards the inactive form of the substrate, which is inaccessible to the kinase (Eq. (13) with 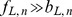). Most of the substrate is in the 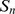 state and the activity is, accordingly, low (Fig. 4). Rather than impeding phosphorylation of the substrate, the addition of phosphatase now enhances it by shifting substrate into states of phosphorylation that are more accessible to the kinase. As a consequence, the concentration of the sink state 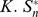 eventually rises and so does the activity, peaking at an intermediate level of phosphatase. As more phosphatase is added, then the expected behaviour occurs: the phosphatase starts winning the competition, the concentration of the sink state 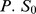 rises, and the activity decreases. The example depicted in Fig. 4, simplified with symmetric rates for the kinase and the phosphatase and a single phosphosite, represents this type of phosphorylation cycle. The system only responds for a certain range of signal and not at both lower and higher concentrations. A similar type of non-monotonic behaviour can also occur in alternative models of phosphorylation cycles, where the kinase and the phosphatase bind to each other [35].

To estimate the size of the parameter space where the non-monotonic behaviour occurs (with 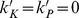), we randomly sampled parameters from a uniform distribution in log-space (therefore assuming equally probable magnitudes). For a system with 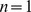, we calculated whether each sampled set of parameters generates a non-monotonic response or not, and we calculated the prominence of the peak for non-monotonic systems, i.e. the difference between the maximal activity of the peak and 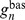 (Eq. (13)). For degrees of saturation between 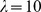 and 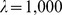, approximately 40% of the parameter space generates non-monotonic behaviour, and its prominence is uniformly distributed between 0 (no peak) and 1 (when the basal level of activity is 0 and the maximal activity, i.e., the height of the peak, is 1).

### Ultrasensitivity does not require allostery

**Figure 5:**
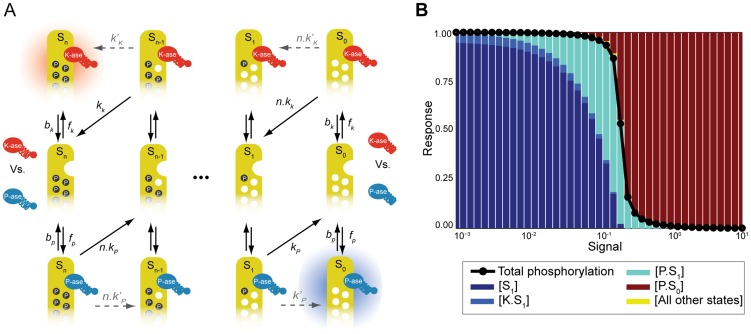
Allostery is not required for ultrasensitivity and models with steric hindrance where the binding of one enzyme inhibits binding of the other can be highly ultrasensitive. (**A**) A model with steric hindrance. The kinase (red) and the phosphatase (blue) bind either to the same docking site or to different docking sites and block the access of the other enzyme to its docking site. The two sinks, 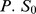 and 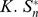, are shown with shadows. The non-distributive catalytic rates (dashed grey arrows) were not allowed in this model. (**B**) Simulation of the response curve (in black) and the concentration of the states of the system (coloured bars) as a function of the signal, i.e., the ratio of phosphatase to kinase for a system, with 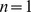, 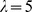, 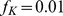 s^−1^, 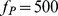 s^−1^ (in units of the inverse total concentration of the substrate), 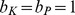 s^−1^, 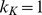 s^−1^, 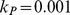 s^−1^. The Hill number is approximately 10.

Our predictions of ultrasensitivity do not depend on allostery. We considered an alternative variation of the model (Fig. 5A), similar to other models that have been proposed [16], [17]. Because distributivity is essential to generate ultrasensitivity, we ignore the non-distributive enzymatic reactions (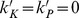, Fig. 5A). The substrate only has one conformational form, to which both the kinase and the phosphatase can bind. They bind to a docking site before modifying the phosphorylation state, but with a constraint: either they bind to the same docking site, and thus only one type of enzyme can be bound at a time, or they bind to different docking sites, but a bound enzyme blocks the access of the opposing enzyme to its docking site via steric hindrance (Fig. 5A). Following the same method as above, we obtain for the model of Fig. 5A (Text S1):
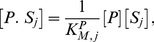
(18)

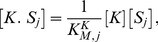
(19)

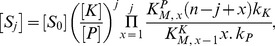
(20)where 
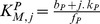
 and 
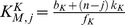
 take the form of the classical Michaelis-Menten constants. The output function is either the fraction of fully phosphorylated states
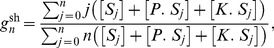
(21)or a weighted average of the phosphorylated states if 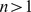. It can be seen that Eqs. (7–10) and Eqs. (18–20) are equivalent in the limit where all allosteric rates are faster than the remaining rates and the allosteric reactions are in quasi-equilibrium (in which case, 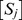 in Eq. (20) equals the sum of both forms of the substrate in the allosteric model). The Hill number thus depends similarly on 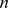. In the symmetric case (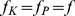, 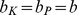 and 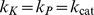), which is analytically solvable, it can be shown the model with steric hindrance also has a Hill number with an upper limit of 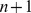. This limit is obtained under the same condition – low dissociation between the enzyme and the substrate in complex – and is also explained by the sequestration of the substrate in two sink states.

This alternative model without allostery shows that it is possible to achieve Hill numbers of magnitude comparable to those predicted by zero-order ultrasensitivity even though 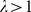. When the enzymes are not symmetric and have different affinities to the substrate and different catalytic rates, then the behaviour of the response can be different between the two models. Parameter sampling and numerical simulations of the model with the allosteric transitions confirms that the upper bound of the Hill number is 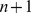 (Fig. S1). The model without allostery but with steric hindrance can, however, generate Hill numbers higher than 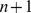 (Fig. S2). Fig. 5B depicts an example of a system with 1 phosphosite (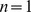) and a Hill number higher than 10, but the ultrasensitivity is dependent on the particular choice of output function. Here we use Eq. (21), and consider a system where the kinase has low affinity to the substrate and the phosphatase has high affinity, but a much slower catalytic rate, 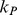. When the input is low, the substrate is primarily in the state 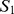. The kinase cannot sequester the substrate in its sink state, but the concentration of phosphatase is too low to bind to the substrate in significant numbers. As the concentration of the phosphatase increases, the complex 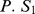 is formed, but the modifying rate 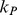 is slow, so the substrate remains in a phosphorylated form. Finally, the addition of phosphatase reaches a critical level where enough unphosphorylated substrate is produced and accumulated in the sink state 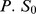 (Fig. 5B). This sharpening of the threshold occurs because the phosphorylation cycle is “jammed” in the 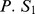 complex. It is only possible if a molecule of substrate is considered active once phosphorylated, rather than in a particular conformational state.

On the other hand, the alternative model of Fig. 5A generates only monotonic response curves: in the absence of signal, the response is maximal (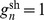); introducing phosphatase in the system reduces the response monotonically.

## Discussion

Here we have shown that a phosphorylation-dephosphorylation cycle with neither the kinase nor the phosphatase saturated can exhibit ultrasensitivity. Both the concentration of the kinase and the phosphatase must exceed that of the substrate and both must first bind to a docking site on the substrate before accessing its phosphosites. We consider either the substrate to be allosteric and in one conformation to bind only the kinase and in the other conformation to bind only the phosphatase or that the binding of one enzyme prevents the binding of another. Ultrasensitivity is mostly generated by sequestration of the substrate either when fully phosphorylated and the substrate is bound by the kinase at its docking site or when fully dephosphorylated and the substrate is bound by the phosphatase at its own docking site. The maximal degree of ultrasensitivity is determined by the number of competitions between the kinase and phosphatase for the substrate. If both enzymes bind simultaneously, i.e., if they have distinct docking sites that remain available when the opposing enzyme is bound, the degree of ultrasensitivity can be substantially reduced and depends on the affinity of each enzyme to forms of the substrate that are either bound by the opposing enzyme or are in conformational states that do not favour binding of that enzyme. In this scenario, each enzyme may no longer be able to sequester the substrate in an inaccessible sink state.

Under the assumptions of our model, namely the rates of binding and unbinding of the enzymes to the docking site in the substrate are independent of the substrate's phosphorylation state, we find maximal ultrasensitivity occurs when the system has a purely distributive scheme, i.e., when the enzymes unbind from the substrate upon catalysing the modification of the phosphorylation state. If, on the other hand, the enzymes do not release the substrate immediately following phosphorylation and dephosphorylation events, then the degree of ultrasensitivity is reduced. Our results on the relative effects of processive and distributive catalytic rates are analogous to those of Dushek *et al.*, who studied ultrasensitivity in phosphorylation-dephosphorylation cycles of membrane-anchored proteins and modelled the diffusion of enzymes towards their substrate explicitly [17]. The authors observed that the system is only ultrasensitive if the enzymatic reactions are diffusion-limited and there exists a refractory period that maintains the enzymes inactive immediately after the catalytic reactions. Those conditions effectively impose a distributive mechanism. Indeed, processive schemes are generally believed to make poor switches [33].

Distributive mechanisms of phosphorylation have been identified in, for example, the phosphorylation of the p42 MAP kinase in *Xenopus* oocytes, and are essential there to generate switch-like responses [36]. In endogenous conditions it is possible that non-distributive and distributive mechanisms coexist within the same phosphorylation-dephosphorylation cycle. Some sites may be phosphorylated or dephosphorylated processively and others distributively, as has been suggested for the phosphorylation of the Pho4 transcription factor in budding yeast [37]. For this type of systems, the Hill number will fall short of its theoretical maximum of 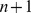, but ultrasensitivity remains possible (Eqs. (15) and (16)).

We expect our model to apply widely. As we have shown, approximately 50% of kinases are expected to have concentrations higher than those of their substrates in budding yeast. Not all copies of the same kinase are however necessarily co-localized with all of their substrates, and so the effective kinase to substrate ratio may be smaller. Having concentrations of the modifying enzymes higher than the substrate could help avoid the slow responses expected when enzymes are saturated [38], as required for zero-order ultrasensitivity. Further, the majority of kinases phosphorylate their substrates multiple times [33] and substrates typically have docking sites for kinases and phosphatases [28], [29], [39]. An example is the pheromone response in budding yeast, where a kinase Fus3 and a phosphatase Ptc1 compete for a substrate Ste5, which has four phosphosites and docking sites for both Fus3 and Ptc1 [16]. As the concentration of extracellular pheromone increases, Ptc1 is recruited to the plasma membrane where it can interact with Ste5 and so its local concentration increases with respect to the kinase Fus3 [16]. As predicted, the degree of ultrasensitivity of the response decreases as the number of phosphosites on Ste5 decreases [16]. Moreover, it has recently been shown the scaffold Ste5 undergoes allosteric transitions, and the kinase Fus3 can only bind one of the conformational states [40], [41].

The ultrasensitivity in our model is generated fundamentally by competitions between the kinase and the phosphatase with the winner sequestering the substrate. Sequestration has been proposed as mechanism for generating ultrasensitivity [8]–[10] and even bistability [42] in phosphorylation cycles and in other systems [43]. Salazar and Höfer proposed a mechanism where enzymes are directly inhibited by their products [8]. In our model, sequestration occurs indirectly because of the existence of docking sites for the enzymes on the substrate and because binding to these docking sites occurs with an affinity independent of the state of phosphorylation of the substrate. This difference is not minor: product-inhibition requires a constraint on the dissociation constants of the substrate-enzyme complexes – the kinase must have low affinity for the phosphorylated state and the phosphatase low affinity for the unphosphorylated state [8]. In our model, ultrasensitivity is independent of whether the enzymes prefer fully modified states of substrate over non-modified states, and Hill numbers higher than 1 can be reached even when the affinities are the same for all states of phosphorylation (Eq. (16)). Liu *et al.* [10] have also proposed a mechanism with regulated degrees of sequestration that is capable of generating ultrasensitivity. In their model, the substrate is sequestered by another protein (a scaffold for example) or translocates to a compartment, depending on the substrate's phosphorylation state. It follows that if, for example, the fully phosphorylated state is sequestered and the phosphatase cannot access it, the system can become ultrasensitive with an upper bound for the Hill number that is equal to the number 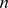 of phosphosites. Liu *et al.* do not model the enzyme-substrate complexes explicitly, but by modelling the formation of those complexes with a two-stage binding process, we have shown that the enzymes themselves can be the agents of sequestration (if each binds to a different allosteric form or if the binding of one enzyme sterically inhibits the binding of the competing enzyme). Additionally, the fully phosphorylated forms of the substrate need not be structurally different from the partially phosphorylated forms, and our model generates ultrasensitivity with an upper bound of 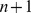. Thus even systems with a single phosphosite can become ultrasensitive.

It is commonly assumed that a simple phosphorylation-dephosphorylation cycle cannot generate ultrasensitivity if the enzymes are not saturated [44]. Our model shows that this assumption is not true if biochemistry is modelled more closely to include docking sites for the enzymes on the substrate. Given that there is little evidence for selection on the ratio of the *in vivo* concentration of kinases to their substrates (as we have shown here), we expect that our mechanism may be common. Phosphorylation-dephosphorylation cycles require energy to operate, but our results emphasize again that “futile” cycles need not be expending energy needlessly but use that energy to generate ultrasensitivity, thus laying the foundation for sophisticated cellular responses such as irreversible switching and oscillations [4].

## Methods

### Estimation of the distributions of enzymes to substrate ratios

The file *BIOGRID-ORGANISM-Saccharomyces_cerevisiae-3.1.87.tab2*, which lists annotated protein-protein interactions in yeast, is available from BioGRID (http://thebiogrid.org/) [18]. We wrote a script to extract the phosphorylation and dephosphorylation interactions and combined the resulting list with the protein expression data of Ghaemmaghammi *et al.* [19].

### Analytical calculations

The detailed calculations of the steady-state concentrations of all states and of the Hill numbers are presented in the Text S1.

### Numerical simulations

The system of ordinary differential equations is simulated using the *Facile* software [45] and MATLAB (The Mathworks, Massachusetts). When multiple simulations to verify the analytical results are referred to in the text, all rates were sampled from log-uniform distributions across six orders of magnitude. The allosteric rates 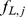 and 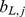 were bound between 1 s^−1^ and 10^6^ s^−1^, while all other rates were bound between 10^−3^ s^−1^ and 10^3^ s^−1^ (where 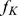 and 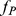 are in units of the inverse total concentration of the substrate).

## Supporting Information

Text S1
**Suporting information.** Section 1 provides a derivation of the equations for the Hill number presented in the main text. Section 2 provides a derivation of a general and compact solution for the state variables at steady state when 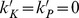 (purely distributive case). Section 3 compares a model of phosphorylation-dephosphorylation cycles, which expends energy and hence has irreversible catalytic reactions, with a Monod-Wyman-Changeux model, which does not expend energy and has reversible catalytic reactions. We show that the Monod-Wyman-Changeux model is not ultrasensitive in response to changes in the concentrations of the enzymes.(PDF)

Figure S1
**Distributions of the Hill number for a non-symmetric version of the allosteric model of** **Figure 2** **.** The Hill numbers were determined by numerical differentiation from 1,000 simulated dose-response curves. For each simulation, the kinetic rates were sampled from log-uniform distributions. The rates 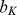, 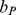, 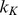 and 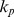 vary between 10^−3^ s^−1^ and 10^3^ s^−1^; the rates 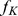 and 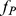 vary between 10^−3^ s^−1^ and 10^3^ s^−1^ (in units of the inverse total concentration of the substrate); the rates 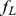 and 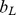 vary between 10^0^ s^−1^ and 10^5^ s^−1^; 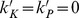 s^−1^. All species are normalised to the total concentration of the substrate, and 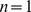. **Top:** 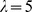. **Bottom:** 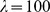.(TIF)

Figure S2
**Distributions of the Hill number for a non-symmetric version of the steric hindrance model of** **Figure 5A** **.** The Hill numbers were determined by numerical differentiation from 1,000 simulated dose-response curves. The purple bars represent Hill numbers higher than 2. For each simulation, the kinetic rates were sampled from log-uniform distributions. The rates 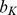, 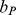, 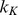 and 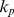 vary between 10^−3^ s^−1^ and 10^3^ s^−1^; the rates 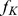 and 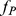 vary between 10^−3^ s^−1^ and 10^3^ s^−1^ (in units of the inverse total concentration of the substrate). All species are normalised to the total concentration of the substrate, and 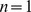. **Top:** 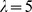. **Bottom:** 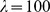.(TIF)

Figure S3
**The allosteric bias enhances ultrasensitivity and increases the number of sink states.** Simulation of the response curve (in black) and the concentration of the states of the system (coloured bars) as a function of the signal, i.e., the ratio of phosphatase to kinase. Here we have 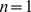, 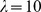, 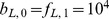 s^−1^, 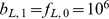 s^−1^, 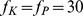 s^−1^ (in units of the inverse total concentration of the substrate), 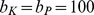 s^−1^, 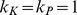 s^−1^, 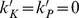. The allosteric bias 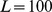 creates an extra sink state, 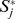, and turns the system ultrasensitive, even while the dissociation rates 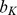 and 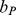 are very fast. The Hill number is approximately 1.7.(TIF)

Figure S4
**Contour plots of the Hill number as a function of the allosteric bias and the relative dissociation rate for** 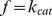 **.** Rows: systems with, from top to bottom, 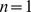, 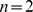 and 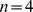 phosphosites. Left column: purely distributive case (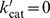); centre column: coexistence of distributivity and non-distributivity (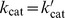); right column: non-distributive case (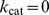). The relative dissociation rate is the ratio of the dissociation rate to the enzymatic rate (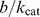 in the left and centre columns; 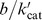 in the right column. The solid black line marks the boundary between subsensitivity (above the line) and ultrasensitivity (below the line).(TIF)

Figure S5
**Contour plots of the Hill number as a function of the allosteric bias and the relative dissociation rate for** 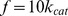 **.** Rows: systems with, from top to bottom, 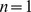, 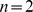 and 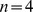 phosphosites. Left column: purely distributive case (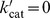); centre column: coexistence of distributivity and non-distributivity (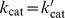); right column: non-distributive case (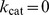). The relative dissociation rate is the ratio of the dissociation rate to the enzymatic rate (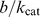 in the left and centre columns; 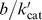 in the right column. The solid black line marks the boundary between subsensitivity (above the line) and ultrasensitivity (below the line). The bottom row corresponds to Fig. 3C in the main text.(TIF)

Figure S6
**Contour plots of the Hill number as a function of the allosteric bias and the relative dissociation rate for** 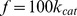 **.** Rows: systems with, from top to bottom, 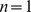, 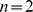 and 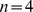 phosphosites. Left column: purely distributive case (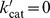); centre column: coexistence of distributivity and non-distributivity (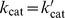); right column: non-distributive case (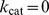). The relative dissociation rate is the ratio of the dissociation rate to the enzymatic rate (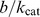 in the left and centre columns; 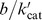 in the right column. The solid black line marks the boundary between subsensitivity (above the line) and ultrasensitivity (below the line).(TIF)
