# Supplementary material for: Ultrasensitivity in Phosphorylation-Dephosphorylation Cycles with Little Substrate
Source: PLoS Comput Biol. 2013 Aug 8;9(8):e1003175. doi: 10.1371/journal.pcbi.1003175 (PMC3738489; doi:10.1371/journal.pcbi.1003175)
Supplement: Text S1 — Suporting information. Section 1 provides a derivation of the equations for the Hill number presented in the main text. Section 2 provides a derivation of a general and compact solution for the state variables at steady state when (purely distributive case). Section 3 compares a model of phosphorylation-dephosphorylation cycles, which expends energy and hence has irreversible catalytic reactions, with a Monod-Wyman-Changeux model, which does not expend energy and has reversible catalytic reactions. We show that the Monod-Wyman-Changeux model is not ultrasensitive in response to changes in the concentrations of the enzymes. (PDF) [file pcbi.1003175.s001.pdf]

Ultrasensitivity in phosphorylation-dephosphorylation cycles with little substrate: Supporting Information

Bruno M.C. Martins, Peter S. Swain

1. Derivation of the equations associated with the main model

From the differential equations that describe the dynamics of the system (Eqs. (1–4) in the main text), the concentrations of the chemical species at steady state are straightforward to obtain: the left hand side of of Eqs. (1–4) becomes zero and we get Eqs. (7–10), reproduced below.

$$\left[P.S_j\right]=\frac{1}{b_P+j\left(k_P+k'_P\right)}\left(f_P\left[P\right]\left[S_j\right]+\left(j+1\right)k'_P\left[P.S_{j+1}\right]\right), \tag{S1}$$

$$\left[K.S_j^*\right]=\frac{1}{b_K+\left(n-j\right)\left(k_K+k'_K\right)}\left(f_K\left[K\right]\left[S_j^*\right]+\left(n-j+1\right)k'_K\left[K.S_{j-1}^*\right]\right), \tag{S2}$$

$$\left[S_j\right]=\frac{1}{b_{L,j}+f_P\left[P\right]}\left(f_{L,j}\left[S_j^*\right]+b_P\left[P.S_j\right]+\left(j+1\right)k_P\left[P.S_{j+1}\right]\right), \tag{S3}$$

$$\left[S_j^*\right]=\frac{1}{f_{L,j}+f_K\left[K\right]}\left(b_{L,j}\left[S_j\right]+b_K\left[K.S_j^*\right]+\left(n-j+1\right)k_K\left[K.S_{j-1}^*\right]\right). \tag{S4}$$

where the rates are as described in the main text. We wish to compute the Hill number of the dose-response curve, which can be defined as proportional to the local sensitivity at the level of input that generates a half response [1] (Eq. (12)):

$$h_n=-2\left(\frac{d\log g_n}{d\log\left[P\right]}\right)_{[P]=[P]_h}. \tag{S5}$$

This is the mathematical definition of the Hill number and can be obtained from a Hill equation. Provided that the type of curve we are analysing is of a similar shape to a sigmoidal Hill curve or a hyperbolic Michaelis-Menten curve, it is similar to other

proxy measures used to estimate ultrasensitivity [2], and it is particularly useful for its analytical tractability.

We keep the kinase levels fixed and vary the phosphatase levels, hence the concentration of the phosphatase normalised to the constant levels of the kinase is the input, and Eq. (S5) is evaluated at the level $[P]_h$ that generates a half maximal response. The response is given by the fraction of active states of the substrate

$$g_n = \frac{\sum_{j=0}^{n}[S_j^*]+[K.S_j^*]}{\sum_{j=0}^{n}[S_j]+[S_j^*]+[P.S_j]+[K.S_j^*]}, \tag{S6}$$

hence we must solve the system of equations S1–S4. We solve the system in an algebra package, such as Mathematica (Wolfram Research, Illinois), constrained to $[K.S_{-1}^*]=0$ in Eqs. (S1, S3) and $[P.S_{n+1}]=0$ in Eqs. (S2, S4) (because both $K.S_{-1}^*$ and $P.S_{n+1}$ do not correspond to any real states). One can, for example, use Eqs. (S1–S4) to first determine $[K.S_0^*]$, $[P.S_0]$, $[P.S_1]$ and $[S_0]$ as a function of $[S_0^*]$; then to use these to determine $[K.S_1^*]$, $[P.S_1]$, $[P.S_2]$ and $[S_1]$ as a function of $[S_0^*]$; and continue until the n-th terms.

If one does the above calculations for $n=1$, $n=2$, $n=3$, etc., and considers an idealised symmetric system when the parameters that govern the activity of the kinase are identical to the equivalent parameters for the phosphatase ($f_K = f_P = f$, $b_K = b_P = b$, $k_K = k_P = k_{\text{cat}}$, $k'_K = k'_P = k'_{\text{cat}}$; and so $[P]_h = K \approx K_T$), as well as identical forward and backward allosteric rates ($b_{L,j} = f_{L,j} = b_L$), one obtains:

$$h_{n=1} = \frac{2b_L k_{\text{cat}} \lambda f + A}{2b k_{\text{cat}} b_L + B}, \tag{S7}$$

$$h_{n=2} = \frac{3b_L k_{\text{cat}} \lambda f + A}{3b k_{\text{cat}} b_L + B}, \tag{S8}$$

$$h_{n=3} = \frac{4b_L k_{\text{cat}} \lambda f + A}{4b k_{\text{cat}} b_L + B}, \tag{S9}$$

…

from whence we conclude:

$$h_n = \frac{(n+1) b_L k_{\text{cat}} \lambda f + A}{(n+1) b k_{\text{cat}} b_L + B}, \tag{S10}$$

where

$A = \lambda f\left(2b_L\left(k'_{\text{cat}} + b\right) + \lambda f\left(k_{\text{cat}} + k'_{\text{cat}}\right)\right)$,

$B = 2b_L b^2 + \lambda f\left(\left(k_{\text{cat}} + k'_{\text{cat}}\right)\left(b_L + \lambda f\right) + b_L k'_{\text{cat}}\right) + b\left(\lambda f\left(2b_L + k_{\text{cat}} + k'_{\text{cat}}\right) + 2b_L k'_{\text{cat}}\right)$.

Rearranging Eq. (S10), one obtains Eq. (15) in the main text. Eq. (S10) has an upper bound of $n+1$ because, under the conditions described in the main text, the first term in the denominator vanishes when b is small.

Eq. (17) in the main text is derived as above for $n=1$, but with nonidentical allosteric rates and disregarding the processive catalytic rates ($k'_k = k'_P = 0$).

2. The general case when $k'_k = k'_P = 0$

If we are simply interested in the case where only distributive reactions occur (diagonal arrows in Fig. 2), then the analytical calculations are more amenable and we can write more elegant solutions for the steady state levels of the states of the system. From Eqs. (1–4) in the main text, we obtain:

$$\left[P.S_j\right] = \frac{1}{K_{M,j}^{P}}[P]\left[S_j\right], \tag{S11}$$

$$\left[K.S_j^*\right] = \frac{1}{K_{M,j}^{K}}[K]\left[S_j^*\right], \tag{S12}$$

$$\left[S_j^*\right] = \left[S_0^*\right]\left(\frac{[K]}{[P]}\right)^j \prod_{x=1}^{j} \left(\frac{K_{M,x}^{P}(n-j+x)k_K}{K_{M,x-1}^{K} x.k_P} \frac{b_{L,x} + \frac{x.k_P}{K_{M,x}^{P}}[P]}{f_{L,x} + \frac{(n-x)k_K}{K_{M,x}^{K}}[K]} \right), \tag{S13}$$

$$\left[S_j\right]=\left[S_j^*\right]\frac{f_{L,j}+\dfrac{(n-j)k_K}{K_{M,j}^K}[K]}{b_{L,j}+\dfrac{j.k_P}{K_{M,j}^P}[P]}, \tag{S14}$$

which have been rewritten as a function of the classical Michaelis-Menten constants $K_{M,j}^P=\dfrac{b_P+j.k_P}{f_P}$ and $K_{M,j}^K=\dfrac{b_K+(n-j)k_K}{f_K}$.

Eqs. (S11) and (S12) are obtained from Eqs. (1) and (2). The general expressions for the concentrations of the substrate states S_j^* and S_j are obtained from the respective ordinary differential equations at steady state:

$$\frac{d}{dt}\left[S_j\right]=0=f_{L,j}\left[S_j^*\right]+b_P\left[P.S_j\right]+(j+1)k_P\left[P.S_{j+1}\right]-\left(b_{L,j}+f_P[P]\right)\left[S_j\right], \tag{S15}$$

$$\frac{d}{dt}\left[S_j^*\right]=0=b_{L,j}\left[S_j\right]+b_K\left[K.S_j^*\right]+(n-j+1)k_K\left[K.S_{j-1}^*\right]-\left(f_{L,j}+f_K[K]\right)\left[S_j^*\right]. \tag{S16}$$

Starting with $j=0$ (denoting unphosphorylated substrate), we use Eqs. (S16) and (S2) to find $\left[S_0\right]$ as a function of $\left[S_0^*\right]$:

$$\left[S_0\right]=\frac{1}{b_{L,0}}\left(f_{L,0}+\frac{n\,k_K}{K_{M,0}^K}[K]\right)\left[S_0^*\right], \tag{S17}$$

and then replace Eqs. (S1) and (S17) into Eq. (S15) obtain $\left[S_1\right]$ as a function of $\left[S_0^*\right]$:

$$\left[S_1^*\right]=\frac{K_{M,1}^P n\,k_K}{K_{M,0}^K k_P}\frac{b_{L,1}+\dfrac{k_P}{K_{M,1}^P}[P]}{f_{L,1}+\dfrac{(n-1)k_K}{K_{M,1}^K}[K]}\frac{[K]}{[P]}\left[S_0^*\right]. \tag{S18}$$

Repeating the steps above for $j=1$, we obtain:

$$[S_1] = \frac{K^P_{M,1} n k_K}{K^K_{M,0} k_P} \frac{[K]}{[P]} [S_0^*], \tag{S19}$$

$$[S_2^*] = \frac{K^P_{M,1} K^P_{M,2} n(n-1) k_K{}^2}{K^K_{M,0} K^K_{M,1} 2 k_P{}^2} \left(\frac{b_{L,1} + \frac{k_P}{K^P_{M,1}}[P]}{f_{L,1} + \frac{(n-1)k_K}{K^K_{M,1}}[K]} \right) \left(\frac{b_{L,2} + \frac{2k_P}{K^P_{M,2}}[P]}{f_{L,2} + \frac{(n-2)k_K}{K^K_{M,2}}[K]} \right) \left(\frac{[K]}{[P]} \right)^2 [S_0^*]. \tag{S20}$$

By repeating this until we reach the n-th terms, Eqs. (S13) and (S14) can be obtained recursively, and used directly to calculate the Hill numbers for purely distributive systems.

For the alternative model of Fig. 5A, Eq. (20) in the main text is obtained recursively from the ordinary differential equation for $[S_j]$ at steady state in the same manner as described above.

3. Comparison to a Monod-Wyman-Changeux model

The Monod-Wyman-Changeux (MWC) model of allostery [3] is a well known mechanism that can generate ultrasensitivity in response to an input. Allosteric proteins are assumed to transition between two conformational states, active (typically called the R, or relaxed, state) and inactive (typically called the T, or tense, state). The transitions are concerted, i.e., if the protein is multimeric, all of its subunits change their conformation simultaneously. In the absence of any input there is an equilibrium bias that favours one of the conformations, e.g., the inactive. Addition of the input reveals a counterbalancing bias: the molecules of input have greater affinity for the active form. Consequently, the presence of input stabilises the active conformation and switches the system on. The dose-response curve of the MWC system can be sigmoidal with the upper bounds of the Hill number being equal to the number of subunits (or the number of binding sites for the input molecules).

One important difference between the MWC system and phosphorylation cycles is that the former does not need to spend energy – all reactions occur in closed thermodynamic cycles and reach chemical equilibrium. We therefore asked what are the consequences of that observation for the dose-response of an MWC system whose allosteric interactions with the molecules of input are governed by enzymes.

Let us consider the following modification of the original MWC model: an enzyme E binds to the substrate in the R state, forming the complexes $E.R_j$. Molecules of input S are then free to bind the substrate, forming the complexes $E.R_{j+1}$ and, if the enzyme unbinds, R_{j+1}. There is an enzyme F, which performs an analogous activity when the substrate is in the T state. When the system is at chemical equilibrium, calculating the levels of the states of the system is straightforward:

$$\left[R_j\right]=\frac{[S]^j}{K_R^{\ j}}\left[R_0\right], \tag{S21}$$

$$\left[E.R_j\right]=\frac{[E]}{K_E}\frac{[S]^j}{K_R^{\ j}}\left[R_0\right], \tag{S22}$$

$$\left[T_j\right]=L\frac{[S]^j}{K_T^{\ j}}\left[R_0\right], \tag{S23}$$

$$\left[F.T_j\right]=L\frac{[F]}{K_F}\frac{[S]^j}{K_R^{\ j}}\left[R_0\right], \tag{S24}$$

where K_R is the dissociation constant of the molecules of input in the R state, K_T is the dissociation constant of the molecules of input in the T state, K_E is the dissociation constant of the enzyme E, K_F is the dissociation constant of the enzyme F, and L is the allosteric equilibrium constant between the R and T states. The dose-response function is given by the proportion of active states

$$f=\frac{\sum_{j=1}^{n}\left[R_j\right]+\left[E.R_j\right]}{\sum_{j=1}^{n}\left[R_j\right]+\left[E.R_j\right]+\left[T_j\right]+\left[F.T_j\right]}\ . \tag{S25}$$

Assuming the concentrations of enzymes and input are much greater than the concentration of substrate and taking $K_E = K_F = K$, we replace Eqs. (S21–S24) into Eq. (S25) to obtain:

$$f = \frac{\left(1+\frac{[S]}{K_R}\right)^n}{\left(1+\frac{[S]}{K_R}\right)^n + L\left(1+c\frac{[S]}{K_R}\right)^n \left(\frac{K+[F]}{K+[E]}\right)}, \tag{S26}$$

where $c = K_R/K_F$. Eq. (S26) is ultrasensitive for signals that affect the ligand S (with Hill numbers of up to n), but not for changes in the enzyme concentrations (if S is fixed), presumably because the action of the enzymes can be easily reverted in energy-free thermodynamic cycles. A phosphorylation-dephosphorylation cycle, as we have studied in the main text, is not so constrained and can generate ultrasensitive behaviour as the ratio of enzyme concentration changes.

References

1. Beard DA, Qian H (2008) Chemical biophysics: quantitative analysis of cellular systems. Cambridge: Cambridge University Press. pp. 84.
2. Goldbeter A, Koshland DE (1981) An amplified sensitivity arising from covalent modification in biological systems. Proc Natl Acad Sci U S A 78: 6840-6844.
3. Monod J, Wyman J, Changeux JP (1965) On the nature of allosteric transitions: a plausible model. J Mol Biol 12: 88-118.
