# Supplementary figures and images for: Ultrasensitivity in Phosphorylation-Dephosphorylation Cycles with Little Substrate
Source: PLoS Comput Biol. 2013 Aug 8;9(8):e1003175. doi: 10.1371/journal.pcbi.1003175 (PMC3738489; doi:10.1371/journal.pcbi.1003175)

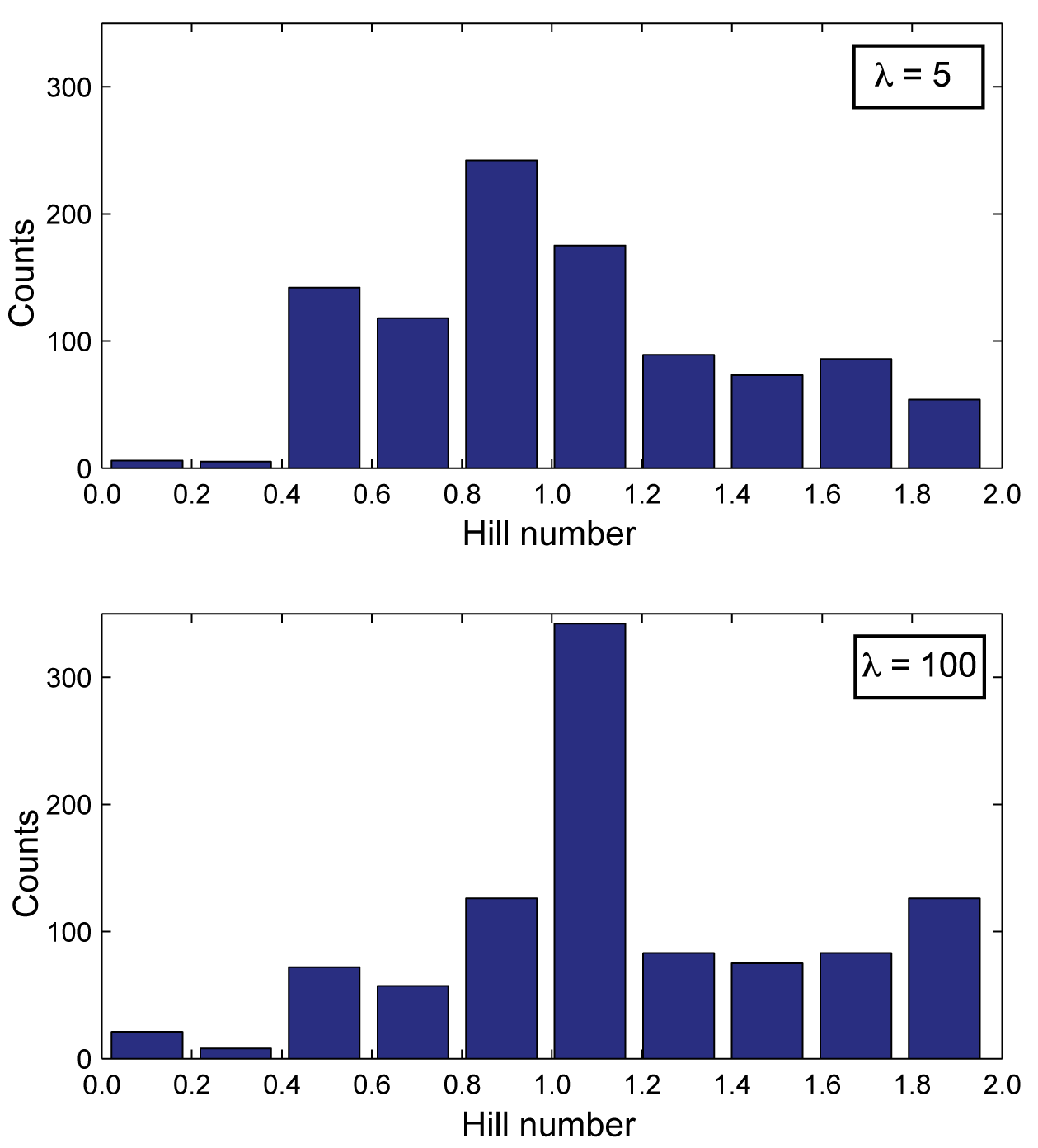

Supplement: Figure S1 — Distributions of the Hill number for a non-symmetric version of the allosteric model of Figure 2 . The Hill numbers were determined by numerical differentiation from 1,000 simulated dose-response curves. For each simulation, the kinetic rates were sampled from log-uniform distributions. The rates , , and vary between 10−3 s−1 and 103 s−1; the rates and vary between 10−3 s−1 and 103 s−1 (in units of the inverse total concentration of the substrate); the rates and vary between 100 s−1 and 105 s−1; s−1. All species are normalised to the total concentration of the substrate, and . Top: . Bottom: . (TIF) [file pcbi.1003175.s002.tif]

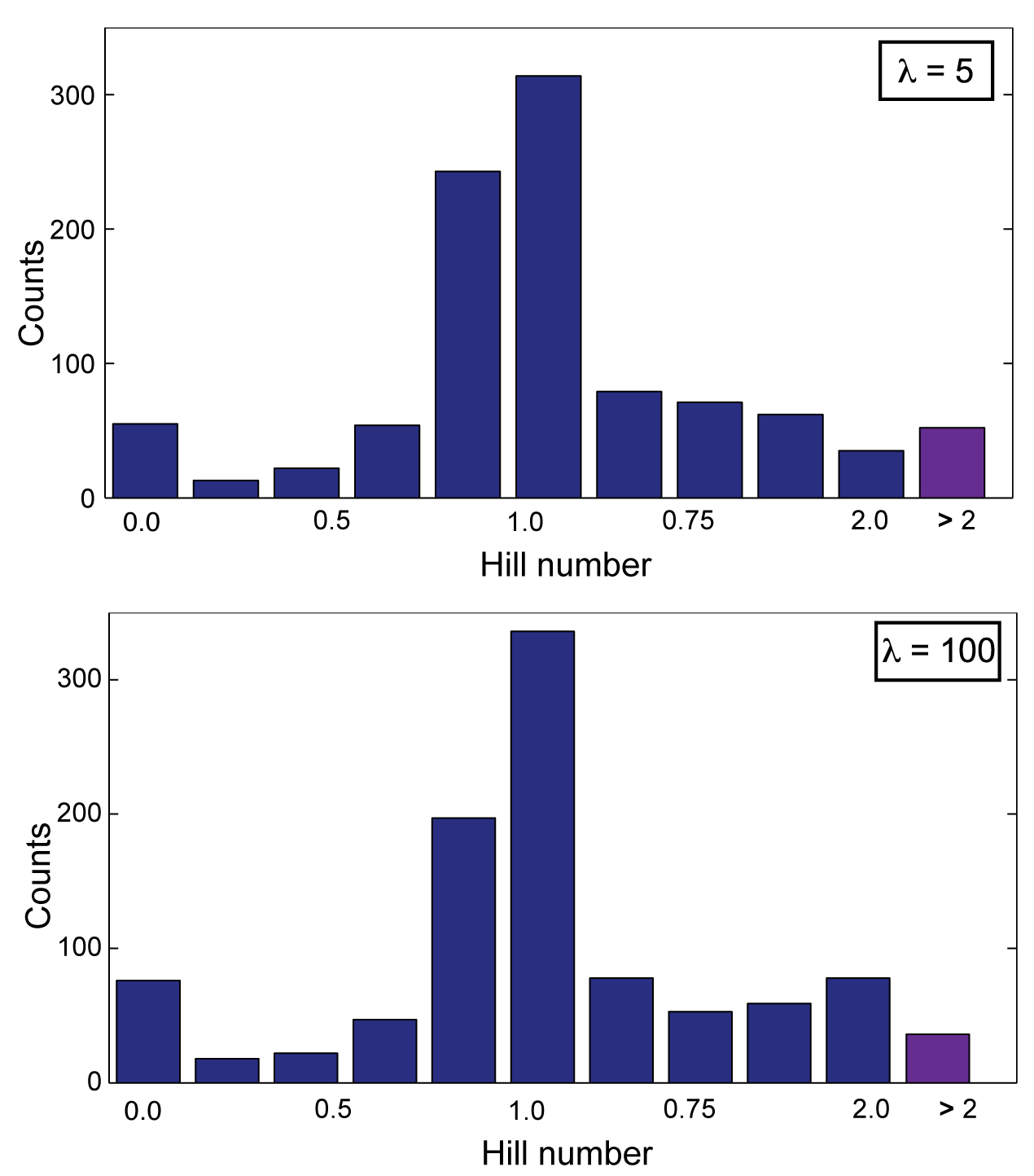

Supplement: Figure S2 — Distributions of the Hill number for a non-symmetric version of the steric hindrance model of Figure 5A . The Hill numbers were determined by numerical differentiation from 1,000 simulated dose-response curves. The purple bars represent Hill numbers higher than 2. For each simulation, the kinetic rates were sampled from log-uniform distributions. The rates , , and vary between 10−3 s−1 and 103 s−1; the rates and vary between 10−3 s−1 and 103 s−1 (in units of the inverse total concentration of the substrate). All species are normalised to the total concentration of the substrate, and . Top: . Bottom: . (TIF) [file pcbi.1003175.s003.tif]

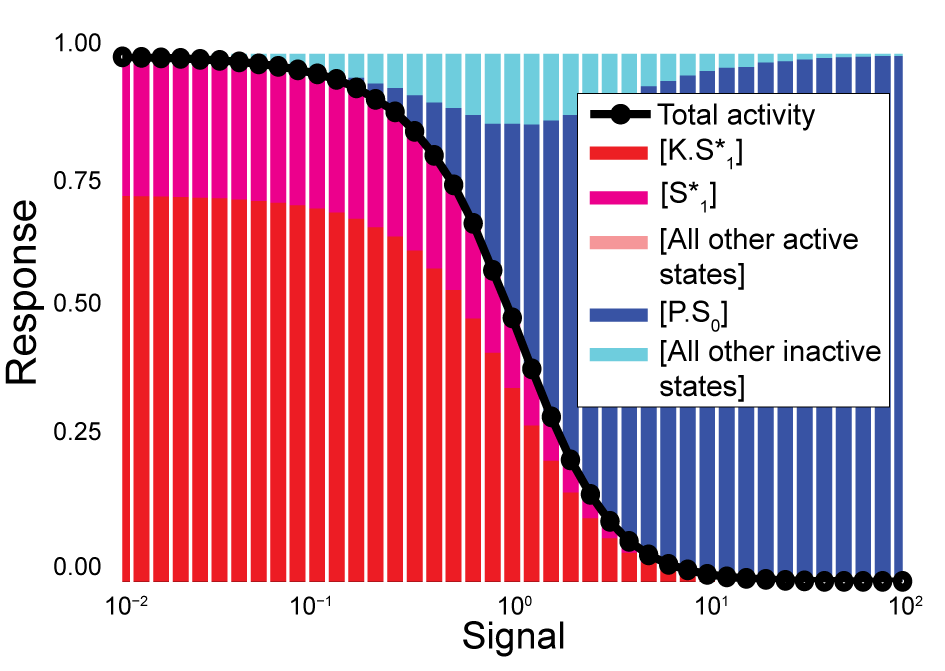

Supplement: Figure S3 — The allosteric bias enhances ultrasensitivity and increases the number of sink states. Simulation of the response curve (in black) and the concentration of the states of the system (coloured bars) as a function of the signal, i.e., the ratio of phosphatase to kinase. Here we have , , s−1, s−1, s−1 (in units of the inverse total concentration of the substrate), s−1, s−1, . The allosteric bias creates an extra sink state, , and turns the system ultrasensitive, even while the dissociation rates and are very fast. The Hill number is approximately 1.7. (TIF) [file pcbi.1003175.s004.tif]

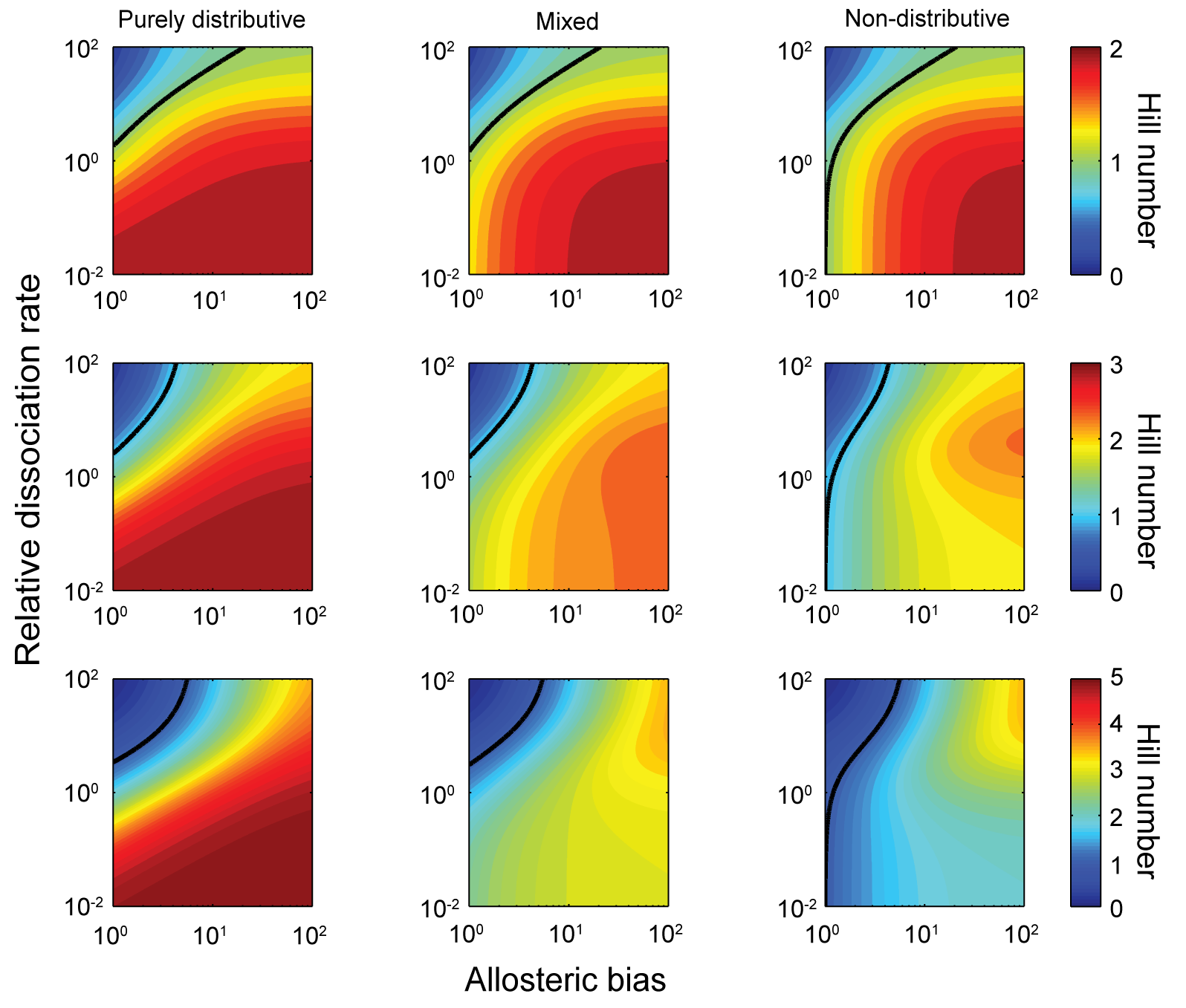

Supplement: Figure S4 — Contour plots of the Hill number as a function of the allosteric bias and the relative dissociation rate for . Rows: systems with, from top to bottom, , and phosphosites. Left column: purely distributive case (); centre column: coexistence of distributivity and non-distributivity (); right column: non-distributive case (). The relative dissociation rate is the ratio of the dissociation rate to the enzymatic rate ( in the left and centre columns; in the right column. The solid black line marks the boundary between subsensitivity (above the line) and ultrasensitivity (below the line). (TIF) [file pcbi.1003175.s005.tif]

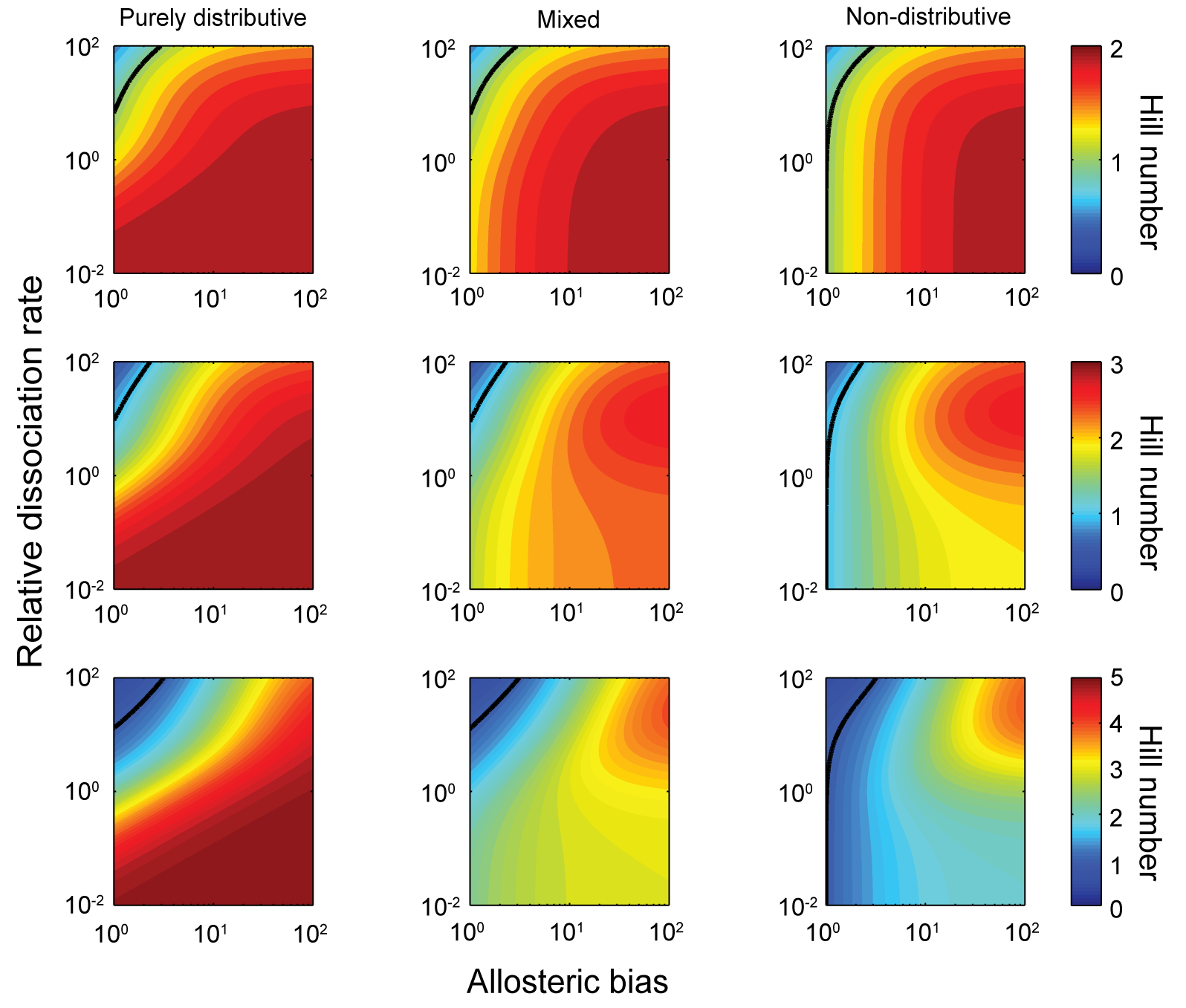

Supplement: Figure S5 — Contour plots of the Hill number as a function of the allosteric bias and the relative dissociation rate for . Rows: systems with, from top to bottom, , and phosphosites. Left column: purely distributive case (); centre column: coexistence of distributivity and non-distributivity (); right column: non-distributive case (). The relative dissociation rate is the ratio of the dissociation rate to the enzymatic rate ( in the left and centre columns; in the right column. The solid black line marks the boundary between subsensitivity (above the line) and ultrasensitivity (below the line). The bottom row corresponds to Fig. 3C in the main text. (TIF) [file pcbi.1003175.s006.tif]

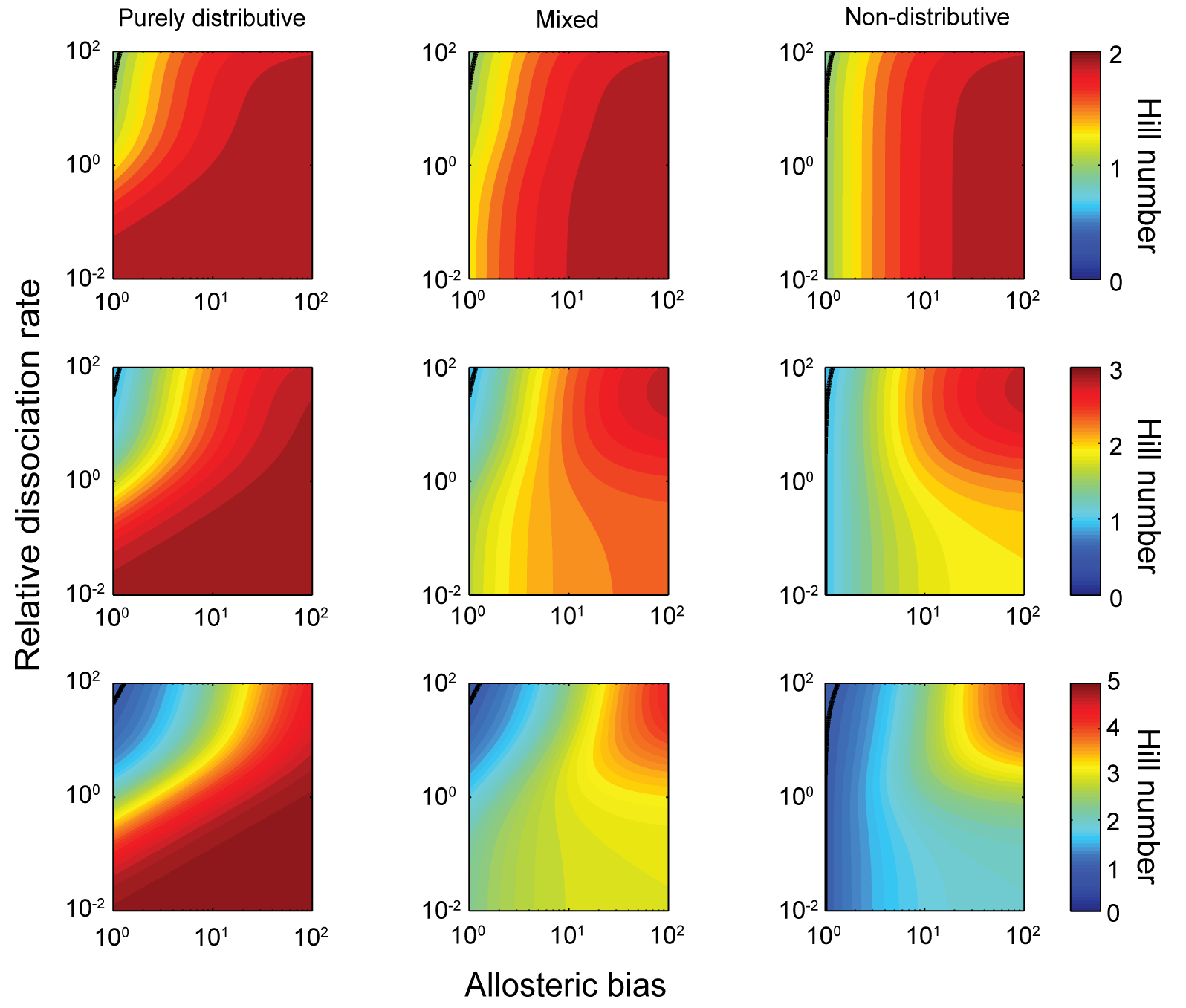

Supplement: Figure S6 — Contour plots of the Hill number as a function of the allosteric bias and the relative dissociation rate for . Rows: systems with, from top to bottom, , and phosphosites. Left column: purely distributive case (); centre column: coexistence of distributivity and non-distributivity (); right column: non-distributive case (). The relative dissociation rate is the ratio of the dissociation rate to the enzymatic rate ( in the left and centre columns; in the right column. The solid black line marks the boundary between subsensitivity (above the line) and ultrasensitivity (below the line). (TIF) [file pcbi.1003175.s007.tif]
